# A novel strategy for the management of cytomegalovirus retinitis in immunocompromised patients using new anti-cytomegalovirus drugs

**DOI:** 10.3389/fmed.2025.1606985

**Published:** 2025-06-11

**Authors:** Yuou Yao, Qiaozhu Zeng, Yaoyao Sun, Enzhong Jin, Jiyang Tang, Yi Cai, Jing Hou, Heng Miao

**Affiliations:** Department of Ophthalmology, Peking University People's Hospital, China and Beijing Key Laboratory of Ocular Disease and Optometry Science, Beijing, China

**Keywords:** novel strategy, antiviral, cytomegalovirus retinitis, letermovir, maribavir

## Abstract

**Objectives:**

This study presents four cases of cytomegalovirus retinitis (CMVR) managed using a novel antiviral strategy, aiming to preliminarily assess its efficacy and safety profile.

**Methods:**

A retrospective chart review was conducted on four patients (seven eyes) diagnosed with CMVR at Peking University People’s Hospital. All patients received oral letermovir or maribavir as the primary treatment. Weekly intravitreal injections of high-dose (6 mg) ganciclovir (IVG) were administered to those with macula involvement or aggressive lesions during the initial treatment. Data on demographics, ophthalmic examinations, laboratory results, and clinical outcomes were analyzed.

**Results:**

All four patients showed clinical and fundoscopic improvement, achieving complete resolution of retinitis. However, one patient developed bilateral retinal detachment requiring vitrectomy. Initial administration of high-dose IVG ensured rapid stabilization of the aggressive or macula-threatening lesions. While subsequent oral antiviral maintenance significantly reduced the need for repeated IVG injections. Both letermovir and maribavir demonstrated excellent tolerability with no adverse events observed.

**Conclusion:**

This novel therapeutic strategy provides safe and effective treatment option for CMVR, particularly promising for patients with complex systemic comorbidities.

## 1 Introduction

Cytomegalovirus (CMV) can lead to severe opportunistic infections in immunocompromised individuals, with retinitis being the most common ocular manifestation (1). Prior to the availability of valganciclovir, CMV retinitis (CMVR) was primarily managed with intravenously administered ganciclovir, foscarnet, and cidofovir (2). Multiple intravitreal ganciclovir injections (IVG) represent another viable therapeutic option for managing CMVR in patients with isolated ocular involvement, serving as the optimal treatment modality when managing patients at risk for systemic ganciclovir toxicity (3). This approach, however, was invasive, expensive, and often poorly adhered to, while failing to address the systemic aspects of the infection (4). The advent of valganciclovir marked a significant shift in CMVR management by offering a fully oral therapeutic regimen. This innovation substantially enhanced treatment adherence and accessibility. However, concerns persist regarding significant adverse effects (AEs), particularly myelosuppression (5).

In recent years, two novel oral anti-CMV agents, letermovir and maribavir, have shown efficacy with minimal adverse effects in post-transplant patients. Letermovir inhibits CMV replication by binding to components of the CMV-terminase complex (6). Maribavir has anti-CMV effects through the inhibition of UL97-mediated phosphorylation of nuclear lamin A/C (7). Although off-label use of letermovir had been reported for patients with CMVR, showing excellent outcomes (8), to date, the therapeutic application of maribavir/letermovir in CMVR remained underexplored as a primary intervention. Here, we present four cases that were primarily managed with letermovir and/or maribavir, aiming to propose a novel and simplified antiviral strategy for patients with CMVR.

## 2 Methods

A retrospective chart review was conducted on patients diagnosed with polymerase chain reaction (PCR)-confirmed CMVR at Peking University People’s Hospital between August 2022 and August 2024, who were treated with letermovir or maribavir as primary therapy. Diagnosis was based on a history of immunosuppression, clinical and fundoscopic findings, positive CMV DNA in aqueous humor, and exclusion of alternative etiologies such as syphilis, tuberculosis, and toxoplasmosis. Data on patient demographics, ophthalmic examinations, laboratory results, clinical outcomes, and adverse events were collected and analyzed. Institutional Review Board approval was obtained by Peking University People’s Hospital (2024PHB 188–01).

All patients received letermovir or maribavir as primary therapy. Within China’s current pharmaceutical regulatory framework, letermovir holds formulary listing status on the 2023 National Reimbursement Drug List, while maribavir remains excluded from both the National Medical Products Administration (NMPA) approval roster and National Reimbursement Drug List (NRDL) formulary, requiring acquisition via cross-border procurement channels. This pharmacoeconomic landscape necessitated implementation of institutional therapeutic algorithms prioritizing letermovir based on multilevel accessibility assessments for most patients.

High-dose IVG (6 mg/0.05 mL) was applied weekly to patients presenting with fovea involvement or aggressive lesions (lesions located within the vascular arcades, in close proximity to the macular region) during initial treatment. Upon a substantial reduction in the activity of the lesion, indicated by sharpening of lesion margins and a transition from vivid coloration to a more attenuated appearance, IVG was discontinued. Subsequently, oral letermovir or maribavir was administered as monotherapy until complete resolution of the lesion was achieved.

## 3 Results

The study included seven eyes from four patients (3 males, 1 female) with ages ranging from 9 to 61 years. The median follow-up duration was 12.75 months (range: 4–24 months). Three patients had bilateral CMVR, while one had unilateral involvement. Clinical data were summarized in Table 1. Patients 1 and 2 had a history of allogeneic hematopoietic stem cell transplantations (allo-HSCT). Patient 3 received chemotherapy for acute lymphoblastic leukemia (ALL). Patient 4 had a history of renal transplantation. All four patients in our cohort had confirmed CMVR, proven by positive CMV DNA in aqueous humor.

**Table 1:** Clinical features and outcomes for 4 patients.

Clinical features and outcomes	Patient 1	Patient 2	Patient 3	Patient 4
Age (yrs)	61	13	9	29
Gender	Male	Female	Male	Male
Eyes involved	Bilateral	Bilateral	Left	Bilateral
Relevant history	Allo-HSCT for ALL	Allo-HSCT for aplastic anemia	ALL chemotherapy	Renal transplantation
Initial CMV DNA load in aqueous humor	OD 5.01 × 10^4^ copies/ml OS 1.04 × 10^4^ copies /ml	OD 2.3 × 10^3^ copies /ml OS 3.08 × 10^4^copies /ml	OS 4.2 × 10^3^copies /ml	OD 2.48 × 10^2^ copies /ml OS 1.95 × 10^3^ copies /ml
Visual acuity at CMVR diagnosis	OD 20/200 OS 20/20	OD 20/200 OS 10/200	OD 20/20 OS 20/60	OD 20/40 OS 20/20
Duration of follow-up	4 months	7 months	16 months	24 months
Novel anti-CMV agents	Maribavir and Letemovir	Maribavir and Letermovir	Letermovir	Letermovir

CMV, cytomegalovirus; CMVR, cytomegalovirus retinitis; allo-HSCT, allogeneic hematopoietic stem cell transplantation.

**Figure 1:**
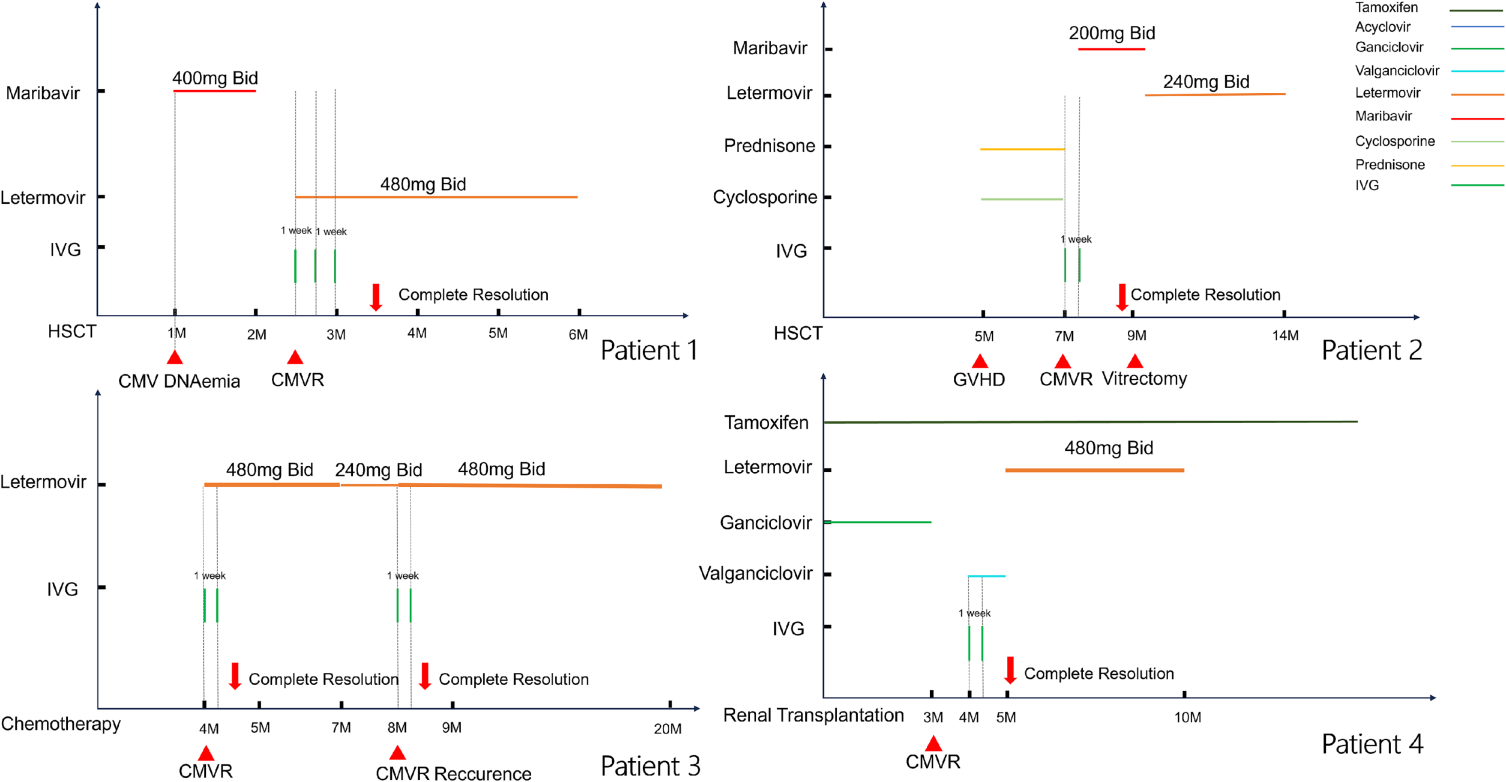
Clinical course of the four cases. The horizontal lines above each graph represent the period on systemic therapy with each agent. Vertical tick marks indicate intravitreal doses.

All patients exhibited both clinical and fundoscopic improvement, leading to the complete resolution of retinitis. Initial high-dose IVG ensured rapid stabilization of the lesions and prevented further vision loss. However, Patient 2 developed bilateral retinal detachment, which required vitrectomy. Figure 1 illustrates the clinical course of the four cases.

### 3.1 Case presentations

**Figure 2:**
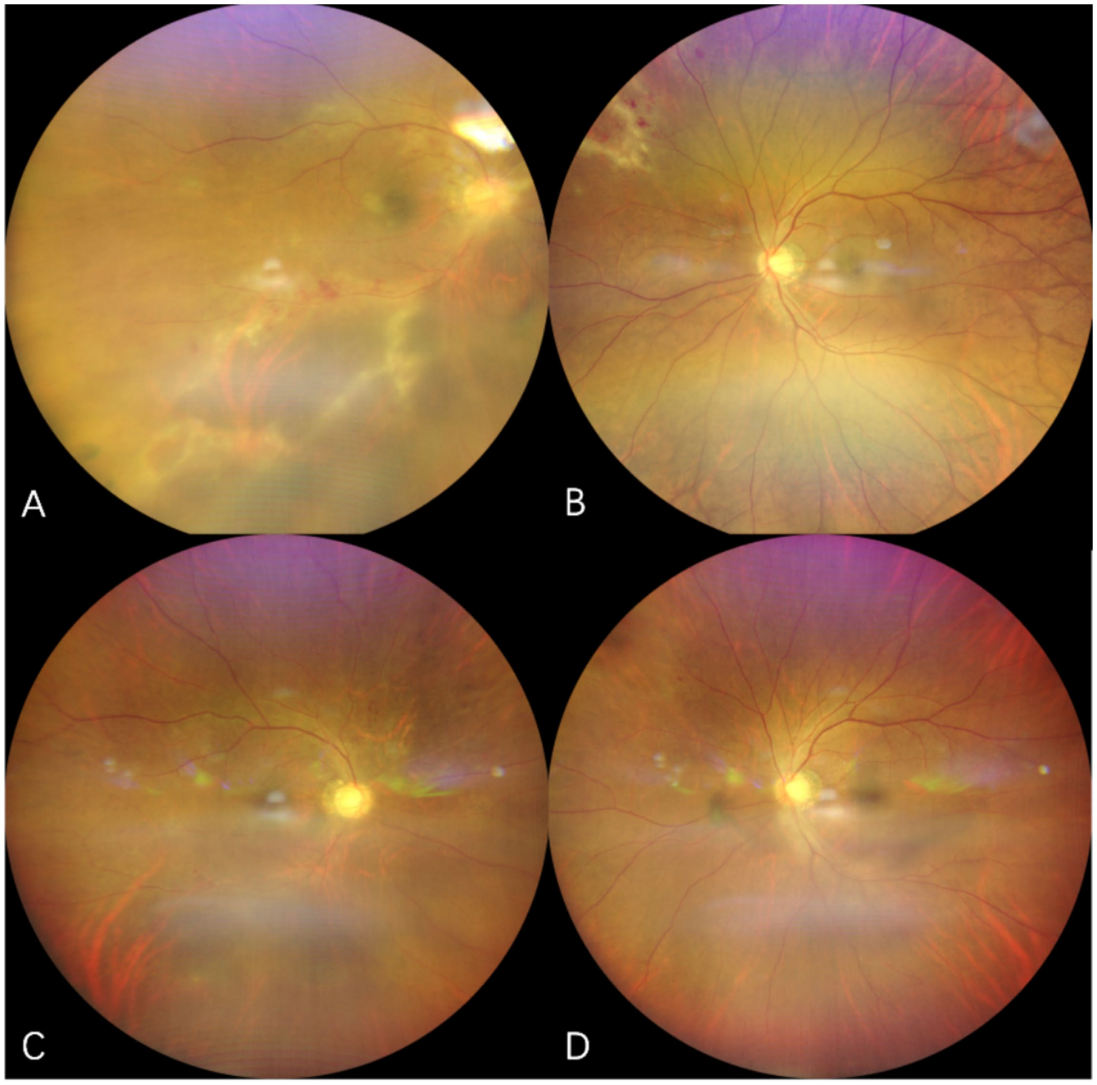
Fundus photograph of case 1. The upper row of pictures **(A,B)** showed initiation of CMVR, revealing yellowish-white retinal necrosis and superficial hemorrhage. The lower row of pictures **(C,D)** showed regression of CMVR after use of letermovir for 6 weeks.

Case 1: A 61-year-old male presented with blurred vision in both eyes for 2 weeks after allo-HSCT 2 months ago due to ALL. He had been administered maribavir 400 mg twice daily for the treatment of systemic CMV infection for 1 month. At the time of assessment, the blood CMV DNA level was quantified at 1.93 × 10^4^ copies/ml. Visual acuity was 20/200 in the right eye and 20/20 in the left eye. Slit-lamp biomicroscope did not reveal aqueous or vitreous cells in either eye. Fundus ophthalmoscopy revealed yellowish-white retinal necrosis and superficial hemorrhage in the peripheral retina (Figures 2A,B). Aqueous humor analysis revealed CMV DNA levels of 5.01 × 10^4^ copies/ml and 1.04 × 10^4^ copies/ml in the right and left eyes, respectively. Whilst awaiting aqueous humor analysis, the lesions exhibited rapid progression. Oral letermovir 480 mg twice daily combined with three IVGs weekly were prescribed, and lesion progression improved dramatically. Following systemic CMV clearance, the patient was followed up for an additional 3 months. Complete resolution of CMVR was achieved within 1 month with no recurrence observed during follow-up (Figures 2C,D), and no AEs were observed.

**Figure 3:**
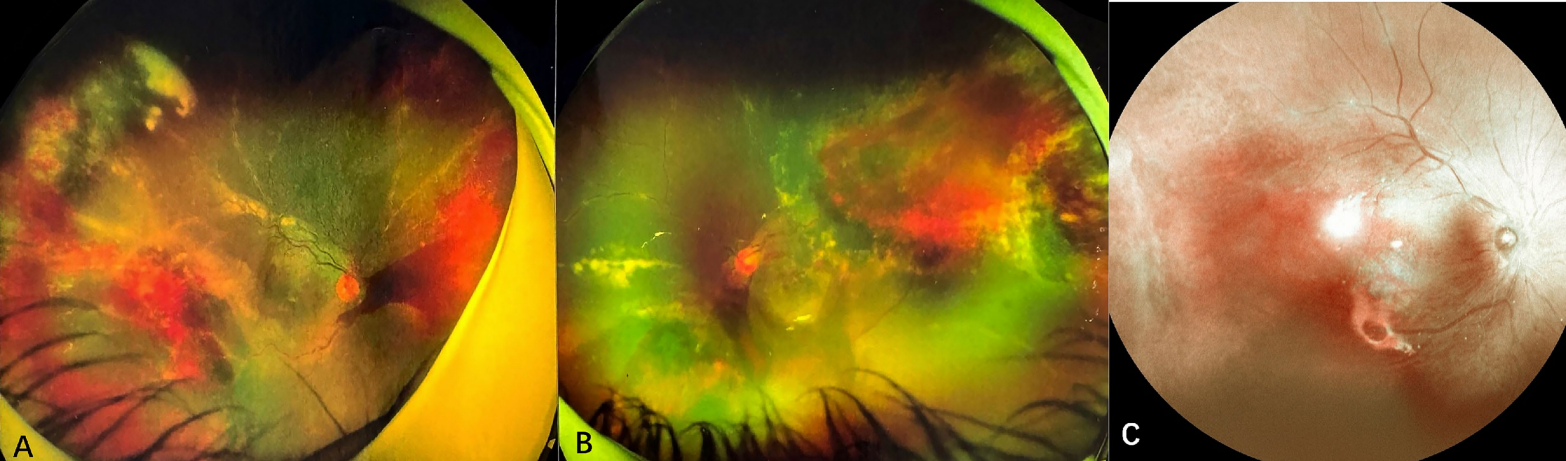
Fundus photographs of case 2. **(A,B)** show the fundus of the right eye and left eye at the time of CMVR diagnosis with characteristic “ketchup cheese retinopathy” appearance. **(C)** lesions in the right eye were completely scarred and inactive after 2 months after the vitrectomy.

Case 2: A 13-year-old female who underwent allo-HSCT for aplastic anemia was given oral corticosteroids and cyclosporine for suspected chronic graft-versus-host disease (GVHD) since month 5 after transplantation. Two months later, she experienced bilateral visual loss and was diagnosed with CMVR (Figure 3). Due to the aggressive nature of the lesion and its involvement of the macula, the treatment included maribavir 200 mg twice daily, considering the low weight of 30 kg, combined with bilateral IVGs (2 sessions with 1 week apart). Although lesions improved rapidly, rhegmatogenous retinal detachment occurred 2 months later, requiring pars plana vitrectomy in both eyes. During the surgery, lesions in both eyes were found to be completely quiescent. The patient was subsequently prescribed with letermovir 240 mg twice daily for the following 5 months. No AEs were observed. Currently, both eyes are filled with silicone oil.

**Figure 4:**
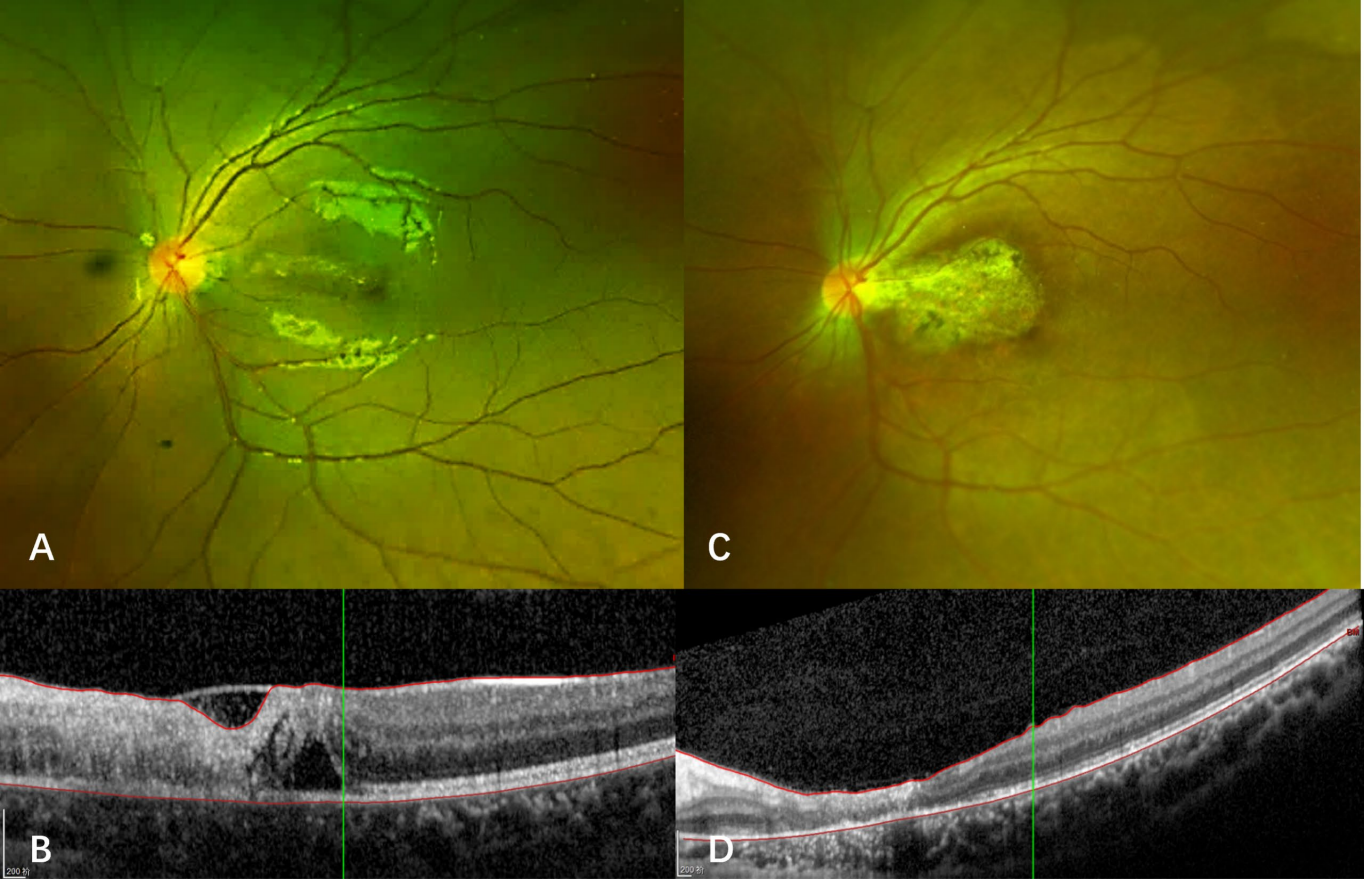
Fundus photograph and optical coherence tomography of case 3. **(A)** At the onset of CMVR, a wedge-shaped area of yellowish-white retinal necrosis is observed between the fovea and optic disc. **(B)** Optical coherence tomography (OCT) reveals thickening, destruction of all retinal layers, and the presence of subretinal fluid. **(C)** The macular lesion regressed after reinstitution of letermovir. **(D)** OCT shows macular atrophy after retinitis resolved with treatment.

Case 3: A 9-year-old male with a history of ALL, who experienced 4 months of chemotherapy, presented with progressive vision loss in the left eye over 1 month. Initial examination showed visual acuity of 20/20 in the right eye and 20/60 in the left eye. Slit-lamp biomicroscope revealed cells (1+) in bilateral aqueous and vitreous. Fundus ophthalmoscopy identified a wedge-shaped area of yellowish-white retinal necrosis between the fovea and optic disc (Figure 4A). OCT revealed full-thickness retinal disruption and subretinal fluid accumulation (Figure 4B). Aqueous humor analysis confirmed CMV DNA at 4.2 × 10^3^ copies/ml. Treatment with letermovir 480 mg twice daily (based on patient weight of 50 kg) and weekly IVG injections (6 mg/0.05 mL) for 2 times led to lesion regression after 1 month. At 3 months, the patient voluntarily reduced letermovir to 240 mg twice daily, which resulted in a recurrence of CMVR with lesion enlargement and a decline in visual acuity to 20/100. Reinitiating letermovir at 480 mg twice daily, in conjunction with another 2 weekly IVGs, led to complete resolution of the lesion (Figures 4C,D). Letermovir 480 mg twice daily was given during the subsequent 1-year follow-up period. No recurrence or AEs were observed.

**Figure 5:**
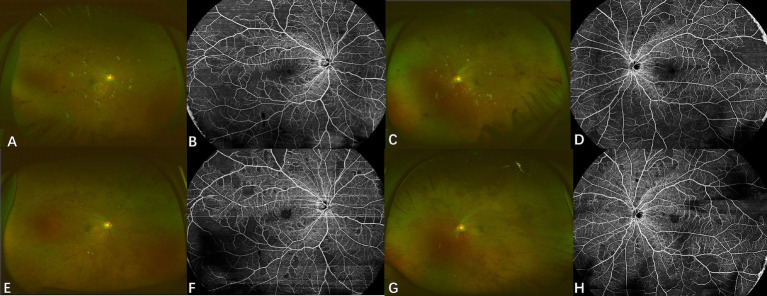
Fundus photograph and optical coherence tomography angiography of case 4. Upper row: Fundus photographs **(A,C)** at diagnosis display multiple cotton wool spots and hemorrhages with a circular pattern along the retinal vessels. Swept-source OCTA images **(B,D)** show retinal non-perfusion in both eyes. Lower row: Fundus photographs **(E,G)** show significant absorption of hemorrhages and cotton wool spots after treatment with letermovir for 4 months. Corresponding swept-source OCTA images **(F,H)** indicate no appreciable expansion of retinal non-perfusion areas after treatment.

Case 4: A 29-year-old male kidney transplant recipient with IgA nephropathy-related renal failure, who had maintained on immunosuppression therapy with tamoxifen for four months, presented with progressive bilateral vision loss after discontinuing oral ganciclovir prophylaxis. Initial visual acuity was 20/40 (right eye) and 20/20 (left eye). Fundus examination revealed multiple cotton wool spots and hemorrhages that distributed along retinal vessels (Figures 5A,C). Swept-source optical coherence tomography angiography (OCTA) showed bilateral extensive retinal nonperfusion (Figures 5B,D). Blood CMV DNA load was 3.66 × 10^5^ copies/ml. CMV DNA was also found at 2.48 × 10^2^ copies/ml and 3.1 × 10^2^ copies/ml in the right and left eye, respectively, and further increased to 8.48 × 10^2^ (right eye) and 1.95 × 10^3^ copies/ml (left eye) one month later, confirming bilateral CMVR. Letermovir 480 mg twice daily was initiated, which led to lesion regression without the need for additional intravitreal injections. Letermovir was continued for another 5 months with no AEs observed. No recurrence or enlargement of retinal nonperfusion area were reported during the 15-month follow-up (5E-5H).

## 4 Discussion

Historically, the systemic or local administration of anti-CMV agents, such as ganciclovir, valganciclovir, cidofovir and foscarnet, has demonstrated efficacy in managing CMVR (9). Intravenous ganciclovir treatment is costly due to hospitalization requirements. Oral ganciclovir is also primarily constrained by two pharmacokinetic challenges: limited gastrointestinal absorption and extensive hepatic first-pass metabolism. This dual barrier system subjects the drug to significant enzymatic degradation during portal circulation transit, drastically reducing its active drug concentration before reaching systemic circulation, thereby compromising therapeutic effectiveness. Moreover, it struggles to cross important biological barriers, including the blood–brain barrier (BBB) and blood-retinal barriers (BRB) (10). These limitations impede the attainment of vitreous drug levels (marginally exceeding the 50% inhibitory concentration [IC50] for wild type CMV), necessitating high daily doses (4-6 g) that often lead to gastrointestinal side effects, notably diarrhea (10). Moreover, ganciclovir associated myelosuppression may not only prolong immune reconstitution, but also severely compromise adherence to therapy, which typically spans several months to one year. Prolonged ganciclovir administration may also induce CMV resistance, diminishing therapeutic efficacy markedly (11, 12). Compared to oral ganciclovir, valganciclovir, a well-absorbed oral prodrug of ganciclovir, demonstrates superior oral bioavailability and fewer AEs (13). However, it shares many of the same limitations as oral ganciclovir. Moreover, in China mainland, the unavailability of valganciclovir further complicates patient access. Its high cost and lack of health insurance coverage impose an additional financial burden on patients (14).

Given these limitations, IVG is regarded as a method capable of achieving adequate vitreous concentrations while circumventing systemic AEs. However, current CMVR treatment strategies, whether conventional or modified IVG regimens, face substantial challenges. These challenges include the need for repeated invasive procedures, which can be painful and difficult for patients in poor health to tolerate or adhere to. Pediatric patients, in particular, often require general anesthesia, adding complexity to repeated injections. Moreover, the need for frequent hospital visits makes the process cumbersome and costly, rendering weekly injections impractical for patients with delayed immune reconstitution needing long-term maintenance therapy. Furthermore, this approach is ineffective in addressing concurrent extraocular CMV infections and/or CMV diseases and does not benefit the contralateral eye.

The advent of innovative antiviral agents, notably letermovir and maribavir, heralds a promising era of fully oral regimens for managing CMVR. Letermovir targets the viral terminase complex, a heterotrimeric structure comprising pUL56, pUL51, and pUL89. This complex plays a crucial role in cleaving concatemeric viral DNA and encapsidating unit-length genomes into nascent virions (15, 16). Letermovir is FDA-approved primarily for CMV prophylaxis post-HSCT, with limited application in CMVR treatment. In our case series, we observed that doubling the typical dosage of letermovir (480 mg twice daily) effectively managed CMVR patients, with favorable tolerability aligning with previous studies (8, 17, 18). Likewise, maribavir, FDA-approved for CMV treatment, is administered at 400 mg twice daily. It acts as a competitive inhibitor of adenosine triphosphate binding to pUL97, a pivotal protein kinase that phosphorylates multiple downstream viral proteins critical to CMV replication (7, 16, 19). Both maribavir and letemovir dosages are halved for pediatric patients due to lower body weight. Both agents offer significant advantages over traditional treatments, simplifying treatment by eliminating the need for hospitalization, repeated surgeries, or intravenous infusions. Consequently, this approach substantially reduces the discomfort and financial burden associated with invasive procedures while enabling prompt treatment initiation upon CMVR diagnosis without delays caused by systemic complications. Additionally, our data suggest that both agents exhibit efficacy against both intraocular and extraocular CMV diseases, while also providing prophylactic protection for the contralateral eye. Furthermore, both letermovir and maribavir show favorable safety profiles, notably avoiding myelosuppression and hepatorenal toxicity. This allows for extended use without compromising immune reconstitution, a duration potentially exceeding one year. As a result, the convenience of these oral regimens enhances patient adherence, particularly for pediatric patients and adults with complex comorbidities.

The *in vitro* IC50 of maribavir typically ranges from 0.01 to 0.3 μM, and its cerebrospinal fluid (CSF) concentration has been reported to be approximately 10–20% of the plasma concentration (7, 20–22). At therapeutic doses (e.g., 400 mg twice daily), the CSF concentration reaches approximately 0.33–1.33 μM (marginally exceeding IC50 of CMV), suggesting potential clinical efficacy. However, the ability of maribavir to cross the blood-ocular barrier (BOB) remains uncertain. In cases of CMVR, a compromised BOB may enhance intraocular drug penetration following oral administration. Nonetheless, while maribavir may achieve sufficient systemic concentrations, obtaining sufficiently high intraocular drug levels to rapidly control ocular lesions poses challenges, particularly in cases with macula involvement or aggressive lesions at initial presentation. This consideration similarly applies to patients receiving letermovir therapy. IVG circumvents these limitations by facilitating direct drug delivery to retinal lesions, achieving intraocular concentrations substantially exceeding the IC50. This ensures a rapid onset of action against both wild-type and resistant CMV strains with elevated IC50 values.

Therefore, an emerging combined therapeutic approach appears to be the optimal strategy. For rapidly progressing lesions, particularly those near or involving macula, initiating treatment with high-dose IVG administered weekly can quickly stabilize lesions. This method prevents visual loss and deterioration by rapidly achieving peak drug concentrations within the vitreous cavity. Typically, CMVR lesions stabilize within approximately 10 days with adequate dosing (23), and two high-dose IVGs treatments are sufficient to cover this critical period. Moreover, concurrent humor aqueous sampling during IVG administration enables effective monitoring of the CMV viral load. The induction phase involves two IVG treatments, which stabilize the lesions. After this, oral antivirals reach their peak intraocular levels, which eliminates the need for further IVG treatments. During the follow-up period, no AEs associated with letermovir or maribavir were observed in any of the cases, further emphasizing the safety profile of this novel approach. This combined approach optimizes CMVR management by balancing rapid action, long-term efficacy, safety, and patient adherence, representing a significant advancement in treatment strategies. Notwithstanding these therapeutic advances, emerging in-vitro and clinical evidence has unambiguously delineated the possibility of letermovir-associated CMV resistance (12, 24). Therefore, clinicians must remain vigilant regarding resistance developed when using these novel antiviral agents. The novel treatment paradigm for CMVR faces several challenges. The current pharmacoeconomic landscape in mainland China reveals a marked disparity in CMV therapeutic accessibility. Maribavir remains constrained by pre-marketing authorization status and exclusion from the NRDL, resulting in dual barriers of limited procurement pathways and prohibitive cost dynamics. Conversely, letermovir’s formulary integration through the 2023 NRDL update has established enhanced formulary accessibility and reduced out-of-pocket expenditure thresholds. This reimbursement-driven therapeutic hierarchy directly informed clinical decision-making in our cohort, with Cases 1 and 2 undergoing protocol-mandated antiretroviral transition from maribavir to letermovir based on institutional drug availability algorithms and cost-efficacy optimization protocols. However, despite its improved accessibility, letermovir still requires supplementary documentation for off-label use in CMVR treatment. Furthermore, there is a paucity of pharmacokinetic data regarding the intraocular half-life, peak and trough concentrations, and volume of distribution for both novel oral antivirals. Consequently, optimal dosing regimens and frequencies for various patient populations, especially for pediatric patients, remain undefined and necessitate further research. Additionally, real-world evidence on the efficacy of these novel antivirals across diverse CMVR patient groups is urgently needed to refine treatment protocols and support evidence-based clinical decision-making.

This study has several limitations. Firstly, it is a retrospective analysis with a small sample size of only four cases. Secondly, as previously mentioned, the majority of patients in this study were treated with letermovir rather than maribavir, limiting our ability to fully elucidate the efficacy of maribavir in treating CMVR. Furthermore, there is currently no consensus on the timing for treatment discontinuation following CMVR resolution. In this study, decisions to terminate treatment were based on clinical presentations during follow-up visits and patient preferences. Lastly, while the concomitant use of IVG was necessary in some patients for rapid disease control, it may have confounded the assessment of the novel oral medications’ effectiveness in CMVR treatment. Future research especially larger, prospective studies should be performed to investigate potential synergistic effects between IVG and oral antivirals, aiming to optimize combination therapies that leverage the strengths of both approaches. Bridging these knowledge gaps will be essential for establishing evidence-based guidelines for integrating novel oral antivirals into CMVR management, ultimately enhancing treatment outcomes for this sight-threatening condition.

In conclusion, the off-label use of novel antiviral agents such as letermovir and maribavir provides an effective and safe therapeutic option for managing CMVR, particularly in patients with delayed immune reconstitution who require extended treatment durations. Furthermore, for patients at risk of macula involvement, initiating early combination therapy with weekly high-dose IVGs alongside novel antiviral agents, can facilitate rapid lesion stabilization and preserve visual function. However, large-scale clinical trials are needed to further validate the efficacy and safety of this therapeutic approach.

## Data Availability

The raw data supporting the conclusions of this article will be made available by the authors, without undue reservation.

## References

[1] Munro M Yadavalli T Fonteh C Arfeen S Lobo-Chan AM. Cytomegalovirus retinitis in HIV and non-HIV individuals. Microorganisms. (2019) 8. doi: 10.3390/microorganisms8010055, PMID: 31905656 PMC7022607

[2] Holland GN. Aids and ophthalmology: the first quarter century. Am J Ophthalmol. (2008) 145:397–408.e1. doi: 10.1016/j.ajo.2007.12.001, PMID: 18282490

[3] Putera I La Distia Nora R Dewi AC Suhada DS Cifuentes-González C Rojas-Carabali W. Antiviral therapy for cytomegalovirus retinitis: a systematic review and meta-analysis. Surv Ophthalmol. (2025) 70:215–31. doi: 10.1016/j.survophthal.2024.11.004, PMID: 39549781

[4] Zandi S Bodaghi B Garweg JG. Review for disease of the year: treatment of viral anterior uveitis: a perspective. Ocul Immunol Inflamm. (2018) 26:1135–42. doi: 10.1080/09273948.2018.1498109, PMID: 30096015

[5] Martin DF Sierra-Madero J Walmsley S Wolitz RA Macey K Georgiou P. A controlled trial of valganciclovir as induction therapy for cytomegalovirus retinitis. N Engl J Med. (2002) 346:1119–26. doi: 10.1056/Nejmoa011759, PMID: 11948271

[6] Marty FM Ljungman P Chemaly RF Maertens J Dadwal SS Duarte RF. Letermovir prophylaxis for cytomegalovirus in hematopoietic-cell transplantation. N Engl J Med. (2017) 377:2433–44. doi: 10.1056/Nejmoa1706640, PMID: 29211658

[7] Sun K Fournier M Sundberg AK Song IH. Maribavir: mechanism of action, clinical, and translational science. Clin Transl Sci. (2024) 17:e13696. doi: 10.1111/cts.13696, PMID: 38071422 PMC10801391

[8] Tsui E Gonzales JA Shantha JG Acharya N Doan T. Letermovir for the management of cytomegalovirus-associated uveitis. Ocul Immunol Inflamm. (2021) 29:169–74. doi: 10.1080/09273948.2019.1662062, PMID: 31638841

[9] Winston DJ Young JA Pullarkat V Papanicolaou GA Vij R Vance E. Maribavir prophylaxis for prevention of cytomegalovirus infection in allogeneic stem cell transplant recipients: a multicenter, randomized, double-blind, placebo-controlled, dose-ranging study. Blood. (2008) 111:5403–10. doi: 10.1182/blood-2007-11-121558, PMID: 18285548 PMC5726327

[10] Tseng A Foisy M. The role of ganciclovir for the management of cytomegalovirus retinitis in Hiv patients: pharmacological review and update on new developments. Can J Infect Dis. (1996) 7:183–94. doi: 10.1155/1996/780831, PMID: 22514437 PMC3327402

[11] Crucio López M Fernández Rivera C Calvo Rodríguez M Alonso Hernández Á. Resistant CMV infection in a transplant patient. Letermovir and withdrawal of immunosuppression. Nefrologia. (2023) 43:661–2. doi: 10.1016/j.nefroe.2023.10.002, PMID: 37951775

[12] Fu L Santhanakrishnan K Al-Aloul M Jones NP Steeples LR. Management of Ganciclovir Resistant Cytomegalovirus Retinitis in a solid organ transplant recipient: a review of current evidence and treatment approaches. Ocul Immunol Inflamm. (2020) 28:1152–8. doi: 10.1080/09273948.2019.1645188, PMID: 31621449

[13] Ude IN Yeh S Shantha JG. Cytomegalovirus retinitis in the highly active anti-retroviral therapy era. Ann Eye Sci. (2022) 7:5. doi: 10.21037/aes-21-18, PMID: 35498636 PMC9053080

[14] Heiden D Saranchuk P. Cmv retinitis in China and se Asia: the way forward. BMC Infect Dis. (2011) 11:327. doi: 10.1186/1471-2334-11-327, PMID: 22115187 PMC3252309

[15] Goldner T Hewlett G Ettischer N Ruebsamen-Schaeff H Zimmermann H Lischka P. The novel anticytomegalovirus compound Aic246 (Letermovir) inhibits human cytomegalovirus replication through a specific antiviral mechanism that involves the viral terminase. J Virol. (2011) 85:10884–93. doi: 10.1128/jvi.05265-11, PMID: 21752907 PMC3187482

[16] Razonable RR. Current perspectives on letermovir and maribavir for the management of cytomegalovirus infection in solid organ transplant recipients. Drug Des Devel Ther. (2024) 18:3987–4001. doi: 10.2147/dddt.S265644, PMID: 39258274 PMC11385360

[17] Turner N Strand A Grewal DS Cox G Arif S Baker AW. Use of Letermovir as salvage therapy for drug-resistant cytomegalovirus retinitis. Antimicrob Agents Chemother. (2019) 63. doi: 10.1128/aac.02337-18, PMID: 30642941 PMC6395918

[18] Veit T Munker D Barton J Milger K Kauke T Meiser B. Letermovir in lung transplant recipients with cytomegalovirus infection: a retrospective observational study. Am J Transplant. (2021) 21:3449–55. doi: 10.1111/ajt.16718, PMID: 34118118

[19] Papanicolaou GA Silveira FP Langston AA Pereira MR Avery RK Uknis M. Maribavir for refractory or resistant cytomegalovirus infections in hematopoietic-cell or solid-organ transplant recipients: a randomized, dose-ranging, double-blind, phase 2 study. Clin Infect Dis. (2019) 68:1255–64. doi: 10.1093/cid/ciy706, PMID: 30329038 PMC6451997

[20] Chou S Marousek G Auerochs S Stamminger T Milbradt J Marschall M. The unique antiviral activity of artesunate is broadly effective against human cytomegaloviruses including therapy-resistant mutants. Antivir Res. (2011) 92:364–8. doi: 10.1016/j.antiviral.2011.07.018, PMID: 21843554

[21] Koszalka GW Johnson NW Good SS Boyd L Chamberlain SC Townsend LB. Preclinical and toxicology studies of 1263W94, a potent and selective inhibitor of human cytomegalovirus replication. Antimicrob Agents Chemother. (2002) 46:2373–80. doi: 10.1128/aac.46.8.2373-2380.2002, PMID: 12121907 PMC127362

[22] Marty FM Winston DJ Rowley SD Vance E Papanicolaou GA Mullane KM. Cmx001 to prevent cytomegalovirus disease in hematopoietic-cell transplantation. N Engl J Med. (2013) 369:1227–36. doi: 10.1056/Nejmoa1303688, PMID: 24066743

[23] Tranos PG Georgalas I Founti P Ladas I. Cytomegalovirus retinitis presenting as vasculitis in a patient with Wegener's granulomatosis. Clin Ophthalmol. (2008) 2:961–3. doi: 10.2147/opth.s4022, PMID: 19668453 PMC2699776

[24] Cherrier L Nasar A Goodlet KJ Nailor MD Tokman S Chou S. Emergence of letermovir resistance in a lung transplant recipient with ganciclovir-resistant cytomegalovirus infection. Am J Transplant. (2018) 18:3060–4. doi: 10.1111/ajt.15135, PMID: 30286286 PMC6263820

